# Electronic Tracking Devices for People With Dementia: Content Analysis of Company Websites

**DOI:** 10.2196/38865

**Published:** 2022-11-11

**Authors:** Jared Howes, Yvonne Denier, Chris Gastmans

**Affiliations:** 1 Centre for Biomedical Ethics and Law KU Leuven Leuven Belgium

**Keywords:** dementia, wandering, electronic tracking devices, bioethics, locators, monitors, surveillance devices, management, technology, care tool, caregiver, device, vulnerable, elderly

## Abstract

**Background:**

Electronic tracking devices, also known as locators, monitors, or surveillance devices, are increasingly being used to manage dementia-related wandering and, subsequently, raising various ethical questions. Despite the known importance technology design has on the ethics of technologies, little research has focused on the companies responsible for the design and development of electronic tracking devices. This paper is the first to perform a qualitative analysis of the ethically related content of the websites of companies that design and develop electronic tracking devices.

**Objective:**

The aim of this study was to understand how companies that design, develop, and market electronic tracking devices for dementia care frame, through textual marketing content, the vulnerabilities and needs of persons with dementia and caregivers, the way in which electronic tracking devices respond to these vulnerabilities and needs, and the ethical issues and values at stake.

**Methods:**

Electronic tracking device company websites were identified via a Google search, 2 device recommendation lists (Alzheimer’s Los Angeles and the Canadian Agency for Drugs and Technologies in Health), and the 2 recent reviews of wander management technology by Neubauer et al and Ray et al. To be included, websites must be official representations of companies (not market or third-party websites) developing and selling electronic tracking devices for use in dementia care. The search was conducted on December 22, 2020, returning 199 websites excluding duplicates. Data synthesis and analysis were conducted on the textual content of the included websites using a modified form of the Qualitative Analysis Guide of Leuven.

**Results:**

In total, 29 websites met the inclusion criteria. Most (15/29, 52%) companies were in the United States. The target audience of the websites was largely caregivers. A range of intertwined vulnerabilities facing persons with dementia and their caregivers were identified, and the companies addressed these via care tools that centered on certain values such as providing information while preserving privacy. Life after device implementation was characterized as a world aspired to that sees increased safety for persons with dementia and peace of mind for caregivers.

**Conclusions:**

The way electronic tracking device content is currently conveyed excludes persons with dementia as a target audience. In presenting their products as a response to vulnerabilities, particular values are linked to design elements. A limitation of the results is the opaque nature of website content origins. How or when values arise in the process of design, development, and marketing is unknown. Therefore, further research should explore the process companies use to identify vulnerabilities, how values are decided upon and integrated into the design of products, and the perceptions of developers regarding the ethics of electronic tracking devices.

## Introduction

### Background

Dementia is an umbrella term for a variety of diseases or conditions that cause progressive loss of cognitive function to the extent that daily life and independence become impaired [1]. Persons with dementia will increasingly come to rely on others for their needs and will typically require some form of institutionalized care [2]. Persons with dementia have expressed their strong desire to remain in their own homes and communities for as long as possible [3,4]. Of the many symptoms of dementia, wandering is a particular problem regarding maintaining a person with dementia safely in their home. Wandering involves movements that have a “frequent repetitive, temporally disordered and/or spatially disordered nature...some of which are associated with eloping, eloping attempts or getting lost unless accompanied” [5]. A person with dementia who elopes while wandering faces a high risk of serious injury or death, with time missing predicting higher likelihood of mortality, making swift recovery paramount [6,7]. Even one incident of wandering can precipitate placement into institutionalized care—despite a person with dementia’s active wish to remain at home [8]. The consequences of wandering extend to those closest to persons with dementia as well; for instance, family, friends, and caregivers may undergo psychological stress, physical fatigue, or other adverse effects during a wandering episode [9,10], and communities often expend large sums of resources to search for a wandering person with dementia [11].

Wandering behavior makes persons with dementia vulnerable to various harms. Vulnerability is a common human condition—to be human is to be vulnerable. This general or common vulnerability is, as ten Have [12] states, “a general characteristic of the human condition.” Our vulnerability is constituted on the realities of our embodied existence, “we are characterized by a general fragility or precariousness because we have a finite, mortal body and because we are unavoidably socially related and dependent on others” [12]. Although all persons are vulnerable, certain individuals, groups, and populations experience a special vulnerability and are “more subject to possible harm and violence than others” [12]. In dealing with dementia and its challenges to physical, psychological, and social capabilities, persons with dementia and their families face a variety of particular vulnerabilities that are exacerbated or lessened by their social context (eg, level of accessible care and social support) [13].

In working to address the vulnerabilities of wandering, technological tools have remained a constant potential panacea. A review found that 83 technologies were being implemented into 26 types of wander management devices, with the most common being electronic tracking devices—also known as locators, monitors, or surveillance devices [14]. Electronic tracking devices are technological artifacts that facilitate the monitoring, locating, or logging of a person with dementia’s temporal location. For example, a watch with a built-in GPS can be used with software to track a wearer’s location in real time or save their location history for later analysis. The idea of using electronic tracking devices to manage dementia-related wandering is not new; however, advances in technology miniaturization and general lowering of technology costs combined with governmental initiatives in assistive technology for older adult care have resulted in an overall increase in electronic tracking devices being brought to market [14-17].

The use of electronic tracking devices is not without ethical concern [18]. Many authors have discussed various concerns related to electronic tracking device use in care practices, including their potential to harm persons with dementia through, for instance, increasing social isolation by reducing contact with caregivers [19,20] or helping facilitate unjust control of persons with dementia [21,22]. More traditional clinical-ethical concerns are also discussed, such as the fraught reality of informed consent within care for persons with dementia who experience wandering symptoms [23] or how a caregiver should best respond to persons with dementia who are resistive to electronic tracking device use [19]. Most normative literature focuses on the use of electronic tracking devices [24], although many ethical questions are related to electronic tracking device design. For instance, privacy hinges on design decisions made during the development process, what data are collected, how they are protected, and who should be informed of a person with dementia’s whereabouts (eg, family, caregivers, unrelated third parties, and law enforcement) [25]. Design-centric concerns also extend to physical specifications such as whether a device should be lockable and unremovable by a person with dementia [18]. On a more global level, design decisions reflect back into concerns regarding the environment and labor conditions [26]. The potential impact technology design has on end users has been widely discussed in the academic literature [27-30] and has seen increased public interest with the growing public awareness surrounding examples such as polarization stemming from social media platforms and bias in machine learning algorithms [15,31-33].

Despite the importance of technology design, relatively little research has been conducted regarding companies responsible for the design, development, and marketing of electronic tracking devices. One avenue of gaining insight into companies’ implicit and explicit ethically related insights is to explore their public positions present within their websites and web-based marketing material. Doing so may reveal how they approach key stakeholders, the problems they face, and the ethical issues involved. In addition, internet use by older adults, in general, has seen steady increases [34], and informal caregivers report turning to the internet to find out information about dementia as a disorder, how they can provide better care, and what professional resources are available to them [35]. As a place of interaction between companies, persons with dementia, and caregivers, a strong motivation exists for undertaking research on how companies present their products on the web. However, to date, little research has focused on this subject. The analysis by Vermeer et al [17] of websites based in the United Kingdom, the Netherlands, and Sweden that market electronic tracking devices for use within dementia care is the only current example. Notably, this study had a relatively wide scope via the inclusion of third-party retail websites (eg, Amazon), and no particular emphasis was placed on ethical questions as an environmental scanning methodology was used for data analysis.

### Objectives

That no study to date has performed an ethical analysis of websites of companies that design and develop their own products is a gap in the literature when viewed in light of the importance of the ethics of technology design, but this gap is more severe when placed in the context of vulnerability. Companies exist within the same social milieu as persons with dementia and their caregivers, one that comprises a multitude of stakeholders, including individuals, companies, and governmental bodies [36]. By placing their products as a means of mitigating the burdens of dementia-related wandering, they insert themselves into the vulnerability of persons with dementia and caregivers, and how they design their products and market them has an effect, whether positive or negative, on the stakeholders involved. With this background, we aimed to gain an understanding of how companies view the vulnerability of persons with dementia and caregivers as well as how they position their products to address these vulnerabilities by performing a qualitative analysis of the written website content for ethically related concepts and themes [37,38]. Hence, our research questions were as follows: how do companies that design, develop, and market electronic tracking devices for dementia care frame, through website textual content, (1) the vulnerabilities and needs of persons with dementia and caregivers, (2) the way in which electronic tracking devices respond to these vulnerabilities and needs, and (3) the ethical issues and values at stake.

## Methods

### Design

We conducted a search for websites inspired by the PRISMA (*Preferred Reporting Items for Systematic Reviews and Meta-Analyses*) statement using the Google search engine and then included websites from electronic tracking device recommendation lists of patient advocacy groups and from 2 recent systematic reviews of wander management technology [39]. We chose to develop the search strategy based on PRISMA so that our search process would be conducted in a transparent and widely recognized manner. Next, we performed a qualitative analysis of the included websites’ content using a modified form of the *Qualitative Analysis Guide of Leuven* (QUAGOL) [40,41].

### Search Strategy

#### Overview

**Figure 1:**
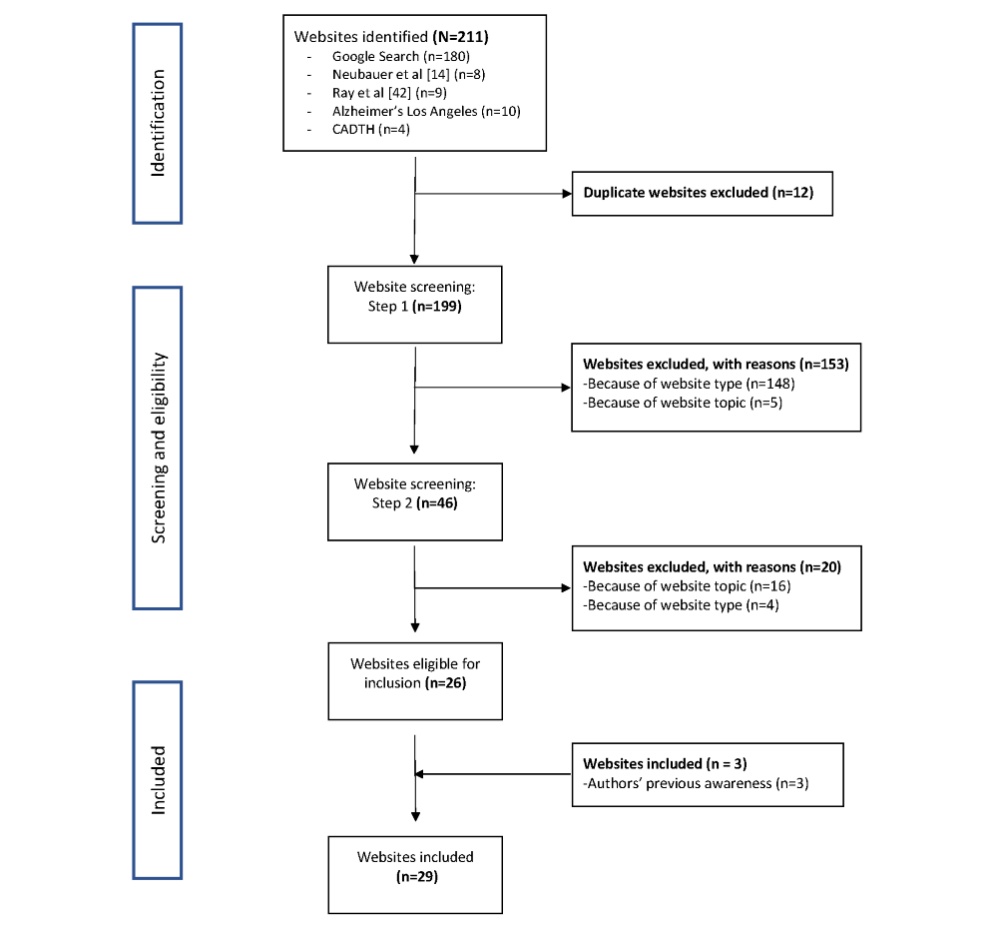
PRISMA (Preferred Reporting Items for Systematic Reviews and Meta-Analyses) flowchart overview of the website identification process [14,42]. CADTH: Canadian Agency for Drugs and Technologies in Health.

A total of 3 sources were used to identify potential websites. The primary source was the results from a Google search conducted according to PRISMA guidelines. The second and third sources consisted of the electronic tracking device recommendation lists provided by *Alzheimer’s Los Angeles* (a patient advocacy organization) and the *Canadian Agency for Drugs and Technologies in Health* and the recent systematic reviews of wander management technology by Neubauer et al [14] and Ray et al [42] (Figure 1). Both the reviews by Neubauer et al [14] and Ray et al [42] were identified after performing a search on PubMed for reviews of electronic tracking devices in the management of dementia-related wandering. The recommendation lists and reviews were included both as additional avenues for identifying potential websites and to serve as a check of the primary Google search.

#### Initial Website Identification

A Google search was the main method for identifying relevant websites. The search engine settings were as follows: the region was set to Belgium, the language was set to English, and 30 results were shown per page. The search string consisted of the following: *“dementia” tracking OR locator device wandering -site:pinterest.com -site:amazon.com*.

During early piloting, it became clear that web pages from Amazon and Pinterest were obscuring relevant results. As any web page from these websites would be excluded based on the inclusion and exclusion criteria (Textbox 1), the advanced search operator *-site:* was used to exclude these web pages from the results [43].

The Google search was conducted on December 22, 2020, returning a total of 250 websites. Of these, only the first 72% (180/250) of websites, or the first 6 results pages, were included as the remaining web pages were increasingly irrelevant or defunct. Other website content analysis studies have limited themselves to 50 results [44,45] or 3 results pages [46].

Additional websites were identified from the electronic tracking device recommendation lists provided by *Alzheimer’s Los Angeles* [47] and the *Canadian Agency for Drugs and Technologies in Health* [48] and by the reviews by Neubauer et al [14] and Ray et al [42]. Relevant references to electronic tracking devices, companies, or websites were followed up with and added to those found with the Google search (Figure 1). The recommendation lists and reviews were included for triangulation and as a check of the Google search results. An indication that the Google search string was not returning relevant websites would be if no websites from the recommendation lists or reviews appeared in the search results. An overlap of 12 websites was found in both the Google search and recommendation lists and reviews, indicating that our search was returning relevant results. After duplicates were excluded, a total of 199 websites were identified.

**Inclusion and exclusion criteria.**

**Inclusion criteria**

- Official website of a company
- Product is advertised for use in managing wandering in persons with dementia
- Product is an electronic tracking device (ETD, which is defined as a technological artifact that facilitates the monitoring, locating, or logging of the temporal location of persons with dementia).
- Website language is English

**Exclusion criteria**

- Market websites, third-party sellers, blogs, and other noncompany websites
- Product is advertised for other population groups (eg, children with autism)
- Product is not an ETD
- Website language is non-English

#### Website Screening

A 2-step process was conducted to screen the websites according to predefined inclusion and exclusion criteria (Textbox 1). The first step was a wide application of the inclusion criteria. A keyword search was conducted on each of the 199 websites to locate references to (1) dementia or Alzheimer disease, (2) tracking device technology, and (3) wandering. If the initial web page did not contain any keywords, a second search was conducted using the advanced search operator *site* in the Google search engine [43]. This operator allows for the identification of every instance of a keyword on a website. For example, by searching for *dementia site:www.Company1.com*, every location in which the word *dementia* appears on any web page within Company1.com would be returned. After the first step, 23.1% (46/199) of the websites generally met the inclusion criteria.

The second step of website screening involved stringently applying the inclusion and exclusion criteria after a close reading of the website content. This involved ensuring that the technology underpinning the device met the definition of an electronic tracking device and that some level of marketing indicated that the device was meant for use in dementia care. A total of 57% (26/46) of the websites met all the inclusion criteria and, with the addition of 3 websites from the authors’ previous awareness, a total of 29 websites were included (Figure 1).

### Data Extraction and Synthesis

Archiving websites as they existed at the time of data extraction is crucial to preserving the broader context in which data are situated and allowing for a precise recall of website content [49,50]. To create an offline archive of the included websites, we used SiteSucker (Rick Cranisky) [51], a computer program for Macintosh OS X that downloads a “[website’s] files to a local folder...while preserving link structure” [52]. This method allows for the preservation of “an entire website in a coherent form” as it was at a particular time [52]. All 29 included websites were archived on February 15, 2021.

Data extraction involved viewing the archived copy of the websites using the Safari web browser (Apple Inc), identifying web pages that contained elements of the inclusion criteria, and exporting them as PDF files.

Characteristics of the companies, websites, and electronic tracking devices were collected via a data collection form (Multimedia Appendix 1). Descriptive statistics were applied to describe the form characteristics of companies, websites, and electronic tracking devices [53]. Textual data on characteristics regarding the content of website information were qualitatively analyzed using a modified form of the QUAGOL [40,41]. Originally developed for qualitative data analysis in original empirical studies, the QUAGOL has also come to be used in analysis of other data sources such as argument-based texts [40,54-57]. The QUAGOL consists of 2 parts, each containing 5 stages. The first part is a thorough preparation of the coding process, and the second part is the actual coding process via a qualitative software program [41]. We modified the QUAGOL in line with previous studies [18,58] by only completing the first 5 stages of the precoding part. The first 5 stages are characterized by an iterative process of diving deeper into the data, with constant movement between the various stages to draw out insights. Thus, although the 5 stages are presented in a sequential order, in reality, one moves between each stage as well as returns to a previous stages throughout the data analysis. In the *first* stage, all researchers thoroughly read the data to gain a holistic understanding of each website. During the *second* stage, 1 researcher (JH) developed initial reports for each website that captured their essence by outlining vital characteristics and key points, with focus being placed on ethically related content. Ethically related content was identified based on themes and concepts present in a previously completed review of argument-based ethics literature [18] and those stemming from the relational care ethics approach as operationalized in the dignity-enhancing care model of bioethics [37,38,59]. Although brief paraphrasing of key quotes was included, initial reports remained as close to the data as possible. The *third* stage entails a movement from initial report to conceptual scheme (Multimedia Appendix 2). Relevant concrete examples from the data are teased out and abstracted into concepts relevant to gaining insight into the research question. It is here that the concepts reported in the results take rudimentary form. To ensure that the concepts were being appropriately and accurately developed, each researcher was given a partially overlapping set of initial reports to independently turn into conceptual schemes. It was found that each author identified the same or similar main concepts, indicating a high level of consensus. During the *fourth* stage, conceptual schemes were tested against the data for accuracy and appropriateness, with researchers rereading the data and initial reports to see if anything was missing, underreported, or in need of modification. The *fifth stage* involved merging all individual schemes into a global scheme through a process of forward-backward movement between each scheme to facilitate the identification of common themes and concepts. It is during this stage that the final conceptual categories were tested and refined before being reported in the Results section of this paper. Throughout the entirety of this work, the research team met regularly to discuss the ongoing process, and an archive of reports, conceptual schemes, and merged scheme drafts has been retained.

## Results

### Form Characteristics of the Companies, Websites, and Electronic Tracking Devices

#### Company Characteristics

First, we detected a general line of company form characteristics—their target audiences, used communication and marketing tools, applied language types, and specific electronic tracking device characteristics.

As for the company characteristics, the headquarters of the included electronic tracking device companies were found throughout North America, Europe, Asia, and Oceania. By company organization, most (23/59, 79%) of the included companies were for profit, a few (3/29, 10%) were nonprofit, and 3% (1/29) were public-private collaborations (Table 1).

**Table 1:** Company characteristics (N=29).

Characteristics			Companies, n (%)
**Country of company headquarters**			
	United States	15 (52)	
	Australia	4 (14)	
	United Kingdom	2 (7)	
	New Zealand	2 (7)	
	Denmark	1 (3)	
	Ireland	1 (3)	
	Finland	1 (3)	
	Spain	1 (3)	
	India	1 (3)	
	Singapore	1 (3)	
**Founding date of company**			
	1980 to 1990	4 (14)	
	1990 to 2000	3 (10)	
	2001 to 2010	5 (17)	
	2011 to 2020	9 (31)	
	Unknown	8 (28)	
**Company type**			
	Nonprofit	3 (10)	
	Private for profit	23 (79)	
	Mix (public-private collaboration)	1 (3)	
	Unknown	2 (7)	

#### Website Characteristics

Predominantly, 3 audiences were targeted by the website content: informal caregivers, formal caregivers (including institutions), and public safety agencies (eg, law enforcement or the fire department; Table 2). Only 3% (1/29) of the companies explicitly targeted persons with dementia themselves. In terms of target users, electronic tracking devices advertised for use with persons with dementia were also often advertised for use with other stakeholder groups such as persons living with various cognitive disabilities (eg, autism) and, to a lesser extent, older adults, children, and employees.

The websites used various tools to share information about their products (Table 2). Most (22/29, 76%) companies had a social media presence, approximately half (12/29, 41%) made use of multimedia resources (eg, video), and all (29/29, 100%) company websites used text-based tools. These information tools were used to convey multiple types of information. Multimedia videos were used to show how a product worked (through guides, overviews, and *how*-*tos*) and why they made a difference (testimonials). All (29/29, 100%) websites used text to describe their products, their benefits, and the advantages they conferred to the target audience. Approximately half (15/29, 52%) contained text-based testimonials. A number (14/29, 48%) of sites contained a blog or news section, which served as a space for posting articles related to dementia, wandering, electronic tracking devices, and other general interest stories, and 10% (3/29) of the websites provided a section for academic and professional resources.

The websites made use of differing types of language to describe electronic tracking devices (Table 2), varying from a rather cold and technical kind of tracking language (eg, tracker device) to more neutral language (eg, monitor and locator) to more warm and personal care language (eg, personal alarm and assistive device). These types of language were not mutually exclusive, and some companies described their products using a mix of language types.

**Table 2:** Website characteristics (N=29).

Characteristics				Companies, n (%)^a^
**Target audience**				
	Informal caregivers			28 (97)
	Formal caregivers (including institutions)			6 (21)
	Public safety agencies			7 (24)
	Persons with dementia			1 (3)
**Target users**				
	**Persons with dementia**			
		Persons with dementia exclusively	6 (21)	
		Persons with dementia nonexclusively	28 (97)	
	**Persons without dementia**			
		Persons living with various cognitive disabilities (eg, autism and Down syndrome^b^)	17 (59)	
		Older adults	9 (31)	
		Other (eg, children, employees, assets, and pets)	6 (21)	
**Information tools**				
	Social media			22 (76)
	No social media			8 (28)
	Multimedia (eg, video or audio)			12 (41)
	No multimedia (eg, video or audio)			12 (41)
	Textual			29 (100)
**Information types**				
	**Multimedia**			
		Testimonials	4 (14)	
		How-tos or guides or product overview	8 (28)	
	**Textual**			
		Product description	29 (100)	
		Testimonials	15 (52)	
		Blog	7 (24)	
		News	7 (24)	
		Academic or professional resources	3 (10)	
**Language used to describe ETDs^c^**				
	Tracking or tracker device			15 (52)
	Tag			1 (3)
	Mobile locator tracking unit			1 (3)
	Location-tracking app			1 (3)
	Monitoring device			2 (7)
	Locator			8 (28)
	Wearable			2 (7)
	Safety app			1 (3)
	Safety device			1 (3)
	Personal emergency location device			1 (3)
	Personal emergency response system			1 (3)
	Assistive device			1 (3)
	Personal alarm			1 (3)
	Wandering alert			1 (3)

^a^The companies may have multiple characteristics.

^b^The companies focus on persons (eg, children) with Down syndrome who do not have dementia.

^c^ETD: electronic tracking device.

#### Electronic Tracking Device Characteristics

With regard to the electronic tracking device characteristics, the most common electronic tracking device form factor was a fob or tag, followed by wristbands (Table S1 in Multimedia Appendix 3). Most electronic tracking devices used a combination of GPS and mobile network technology, with some also including additional technology. Most device prices ranged between US $1 and US $500. Some electronic tracking devices required a subscription to operate, most costing between US $1 and US $50 per month.

### Characteristics Regarding Content of Website Information

Second, we found that the electronic tracking device company websites were largely organized around three overarching dimensions describing (1) the vulnerabilities that persons with dementia and their caregivers have to face, (2) the care tools that electronic tracking devices possess that address these vulnerabilities, and (3) a conception of the world that is aspired to and comes about from using the presented electronic tracking device. In the following sections, we will describe these 3 dimensions in more detail.

#### Expressed Vulnerabilities

##### Overview

During the data analysis process, it became clear that the companies identified a range of vulnerabilities facing both persons with dementia and their formal or informal caregivers. These vulnerabilities stemmed from dealing with dementia-related wandering and from using electronic tracking devices themselves. They can be grouped into the following categories: bodily, psychological, social, existential, moral, and technical vulnerabilities (Table 3).

**Table 3:** Overview of company-identified vulnerabilities (N=29).

Vulnerability			Companies, n (%)^a^	
**Bodily vulnerabilities**				
	**Persons with dementia**			
		Wandering		26 (90)
	**Caregivers**			
		Limited in body		3 (10)
**Psychological vulnerabilities**				
	**Persons with dementia**			
		Loss of independence		26 (90)
		Feeling insecure or unsafe		6 (21)
		Feeling like a burden on family		3 (10)
		Psychological or emotional stress		4 (14)
	**Caregivers**			
		Psychological or emotional stress		21 (72)
		Lack of knowledge		6 (21)
		Loss of independence		5 (17)
**Social vulnerabilities**				
	**Persons with dementia**			
		Erosion of social life (eg, isolation)		3 (10)
	**Caregivers**			
		Erosion of relationship with person with dementia		2 (7)
		Need for external support or help		4 (14)
**Existential and moral vulnerabilities**				
	**Persons with dementia**			
		Erosion in quality of life		8 (28)
	**Caregivers**			
		Faced with difficult decisions		6 (21)
**Technical vulnerabilities**				
	**Persons with dementia**			
		Stigma derived from ETDs^b^		7 (24)
		Physical discomfort from ETDs		5 (17)
	**Caregivers**			
		Exacerbated vulnerabilities from difficult-to-use ETDs		17 (59)

^a^The companies may have multiple vulnerabilities.

^b^ETD: electronic tracking device.

##### Bodily Vulnerabilities

Bodily vulnerability relates to potential physical risks and harms. All (29/29, 100%) companies identified the physical risks related to wandering as the principal vulnerability facing persons with dementia. Becoming lost can lead to serious injury or even death. This vulnerability is the primary reason that companies have developed electronic tracking devices and, in some sense, is the cause of all other identified vulnerabilities. Caregivers’ bodily vulnerabilities were also described, albeit to a lesser extent. The companies pointed to caregivers’ physical limitations (such as increasing fatigue or exhaustion) that can be reached when they are required to monitor, care for, or otherwise attend to a person with dementia at all times.

##### Psychological Vulnerabilities

According to the company websites, the predominant vulnerability facing caregivers is psychological or emotional stress. As persons with dementia experience more severe symptoms of dementia and begin to wander, caregivers are confronted with stress-inducing situations. Fearing for the safety of their loved one, they may feel unconfident in current safety arrangements but lack the knowledge to rectify or improve the situation. As the care needs of a person with dementia grow, caregivers may become increasingly focused on providing care to the exclusion of personal interests or pursuits, making them vulnerable to losing their own independence.

Persons with dementia, too, are psychologically vulnerable, with many companies underscoring loss of independence. This loss can be external in origin through, for instance, limitations placed by caregivers, but it may arise internally as well; persons with dementia who are aware of their potential for wandering may feel insecure or unsafe and, thereby, self-limited. The loss of independence is seen as a contributor to feelings of isolation and loneliness and, as care needs continue to rise, of being a burden upon one’s family. These specific examples coincide with companies alluding to the general psychological or emotional stress that can occur during routine dementia care.

##### Social Vulnerabilities

Various social vulnerabilities were found within the websites’ content. Persons with dementia may experience an erosion in their social life, both because of the course of dementia and as a result of potential restrictions aimed at reducing the physical and psychological risks of wandering. Informal caregivers, too, may see their social lives harmed. As they devote increasing time to care responsibilities, they may set aside their own social engagements and responsibilities; in the case of children caring for parents, life plans may be delayed or changed.

##### Existential and Moral Vulnerabilities

Existential vulnerabilities entail the risk of harms related to one’s existential self and one’s own experience of being able to live a meaningful life. In this regard, the company websites referred to the risk of being institutionalized against one’s wishes, which is closely associated with the potential for the person with dementia to experience a general degradation in quality of life. Moral vulnerability deals with the difficulties surrounding moral decisions. Many companies acknowledged that caregivers are vulnerable when faced with difficult care decisions, often running into difficulties in balancing the aim to keep a person with dementia safe with the wish to maintain a person with dementia’s independence and autonomy in leading their own life.

##### Technical Vulnerabilities

The companies were very cognizant of the new vulnerabilities that electronic tracking devices introduce in persons with dementia and their caregivers. Difficult-to-use technology may exacerbate the psychological and emotional stress of persons with dementia and caregivers. Furthermore, if an electronic tracking device is not able to easily integrate into the daily life or habits of persons with dementia and their caregivers, it may lead them to missing the full benefits of an electronic tracking device or, in a worst-case scenario, outright abandon the technology. For example, “choosing a GPS transmitter designed for shoes is not good if the person with dementia has the habit of taking their shoes off” (company 12). The form factor of the device itself opens persons with dementia up to new vulnerabilities. Physical discomfort may increase agitation or stress in persons with dementia, and they may even injure themselves if they attempt to remove an unwanted or unfamiliar device. Device esthetics may also contribute to the stigmatization of persons with dementia.

#### Care Tools of the Electronic Tracking Device: Addressing the Vulnerabilities

##### Overview

The second overarching dimension that was present in the company websites represents the various ways in which the care tools that electronic tracking devices possess address the aforementioned vulnerabilities. By outlining specific device designs, functions, or capabilities, the companies positioned their electronic tracking devices as the right care tool for helping both persons with dementia and their caregivers address or even overcome their vulnerabilities. Accordingly, we detected several categories of tools that serve as a response, viz providing information, supporting communication, creating communities of care, and focusing on adjusted and user-friendly design (Table 4).

**Table 4:** Overview of electronic tracking device care tools (N=29).

Care tool			Companies, n (%)^a^
**Providing information**			
	High level of information availability	16 (55)	
	Medium level of information availability	4 (14)	
	Low level of information availability	5 (17)	
**Supporting communication**			
	Two-way	8 (28)	
	One-way	3 (10)	
	SOS function	10 (34)	
**Realizing communities of care**			
	Personal care community	5 (17)	
	Formal care community	10 (34)	
**Adjusted and user-friendly design**			
	Easy-to-use design	17 (59)	
	Form factor	11 (38)	

^a^The companies may have multiple care tools.

##### Providing Information

In total, 3 levels of information availability were offered by marketed electronic tracking devices. At the highest level, the companies granted caregivers near-total access to the real-time location of persons with dementia. The motivation behind this decision varies. Information plays an important role in providing peace of mind to caregivers and improving overall care quality by providing caregivers with the tools necessary to keep the person with dementia safe. Increased information about a person with dementia increases a caregiver’s situational awareness and enables them to proactively protect the person with dementia by monitoring, adjusting, and intervening when necessary. In addition, with greater information, direct control is given back to caregivers. Being able to modify various parameters, such as the geographical zone where a person with dementia can walk freely, empowers caregivers in managing their loved one’s condition. Devices marketed toward institutional care settings focused on better resident management, for example, by showing the location of all residents on a facility map.

At the middle level of information availability were devices that allow a caregiver to view a person with dementia’s location only if certain thresholds are met, for instance, when a person with dementia leaves a defined geographical zone, a set ambient temperature is reached, or on a fixed time schedule. The motivation for such a mediated flow of information is based upon a balance between the ethical values of autonomy and bodily integrity. The companies strived to uphold a person with dementia’s privacy and freedom (autonomy) while also attempting to keep them safe (bodily integrity). Company 12 advertised that their product gave persons with dementia “more privacy, greater autonomy and more reassurance.” An additional motivation is the desire to optimize battery capacity, with periodic rather than constant updating increasing time between charges.

The lowest level of information availability consisted of devices whose underlying technology cannot facilitate real-time tracking or localizing. Similar to those in the higher levels, these devices can raise an alarm when a person with dementia leaves a defined geographical zone, with a radio receiver then being used to hone in on the person with dementia. The choice to use this technology centers on producing a simple, reliable, and affordable product. As company 4 stated, “telemetry tracking is not rocket science.” Such technology also comes standard with a very long battery life (≥6 months), meaning caregivers do not need to remember to charge or replace batteries often. An additional benefit of using this technology is that privacy is more readily protected as a person with dementia can only be tracked if the identification number for the receiver is known.

##### Supporting Communication

Many companies whose devices offered high- or middle-level information availability highlighted communication as an important care tool. Two-way communication is a feature that allows a caregiver to speak with a person with dementia as if over the phone, allowing for greater interpersonal connection as well as enabling a caregiver to speak with a person with dementia during an emergency. Often, the person with dementia does not need to do anything as the electronic tracking device automatically answers the call. Another feature in some devices is one-way listening. With this, a caregiver is able to listen in and hear what is happening in a person with dementia’s surroundings. A communication tool unique to the person with dementia is an SOS emergency button. When a person with dementia presses the SOS button, an emergency notification or alert is sent to caregivers or a monitoring service indicating that something is wrong.

##### Creating Communities of Care

Creating communities of care was also present within company website content as an answer to address existing vulnerabilities. The companies built tools into their electronic tracking devices that were geared toward supporting informal caregivers by enabling them to create a personal care community. Some electronic tracking devices are usable only within formal care communities such as institutional care facilities, emergency services (eg, police department and fire department), or local nonprofit chapters. The motivation behind embedding an electronic tracking device into such a community varies. The institutional knowledge cultivated and stewarded by formal care communities may provide unique benefits to persons with dementia and caregivers as enrollment means becoming “a part of a community that is dedicated to their safety and well-being” (company 14). In addition, the companies can ensure that their products are not being misused, for example, using an electronic tracking device on someone without a cognitive condition or medical need.

##### Adjusted and User-friendly Design

The companies emphasized that their products were easy to operate and integrate into daily life. This is seen as important for meeting the needs of persons with dementia and caregivers as complicated and difficult technology may increase stress and frustration, and technology that is hard to integrate into life has a high chance of abandonment. A number of electronic tracking device design elements are marketed as contributing to ease of operation. Software design such as font size or an easy-to-use mobile app or website contribute to easier operation. So, too, does physical design, with companies focusing on the frequency and method in which an electronic tracking device will need to be charged, the shape and feel of the device, and the manner in which it is worn.

A device that a person with dementia will not wear is not useful. Physical form factor, such as comfortability or style, is seen as important for electronic tracking device acceptance by persons with dementia. Sleek, stylish, or discreet devices are also put forward to protect persons with dementia from being stigmatized, experiencing indignity, or becoming targets for criminal activity. Company 13 pointed to the benefits of a small, lightweight, and unobtrusive device: “Don’t embarrass Mom with garish panic jewelry.”

A key point of design for improved integration is customizability. Not all persons with dementia and caregivers have the same vulnerabilities, problems, or lifestyles and, therefore, having an electronic tracking device that can be adapted to suit individual contexts better meets real-world needs. Customization can range from the manner in which an electronic tracking device is worn, the length of time that movement history is stored, frequency of updates or notifications, defined geographic “safe” zones, and more. This customizability also extends to formal care institutions, with many companies offering to tailor products and services to the needs of particular institutions.

#### A World Aspired to

##### Overview

The third overarching dimension that was present in the company websites represents a conception of the world that is aspired to and comes about from using the presented electronic tracking device. Many companies offered a vision of life after the implementation of their product. This “world aspired to” is how the companies anticipated their electronic tracking devices would change the daily lives of persons with dementia and their caregivers (Table 5).

**Table 5:** Anticipated benefits and recognized limitations of electronic tracking device (ETD) use (N=29).

				Companies, n (%)^a^
**Anticipated benefits**				
	**Persons with dementia**			
		Increase safety	23 (79)	
		Maintain or increase independence	11 (38)	
		Improve psychological or emotional state	9 (31)	
	**Caregivers**			
		Increase peace of mind	21 (72)	
		Increase knowledge	9 (31)	
		Enable higher-quality care	7 (24)	
		Increase caregiver independence	4 (14)	
	**Both persons with dementia and caregivers**			
		ETD is affordable and cost-effective	6 (21)	
		Improve interpersonal relationship	3 (10)	
**Recognized limitations of ETDs**				
	Technical limitations			7 (24)
	Role and fit limitations			6 (21)
	Price limitations			6 (21)
	Ethical limitations			4 (14)

^a^The companies may appear in multiple categories.

##### Bodily Safety and Psychological Peace of Mind

For persons with dementia, the most saturated aspiration put forward by the companies was increased bodily safety. The implementation of electronic tracking devices will result in reducing the risks associated with wandering as a person with dementia can be easily kept track of and located during an emergency. As a result of bolstered bodily integrity, the companies envisioned a range of new benefits for persons with dementia. Psychologically and emotionally, persons with dementia will see their independence maintained or increased. As company 16 described, “[t]he Safer Walking GPS Locator exists to provide a safeguarding measure so that a person living with dementia can be encouraged to continue to get outside and walk, live well and enjoy social interaction and independence.” In addition, persons with dementia may experience a general improvement in their psychological and emotional state being confident in their situation, feeling safer or more secure, and not having “the unpleasant feeling of being a burden or strain on their relatives” (company 12).

In the same way, the companies aspired to a world where a caregiver choosing to integrate an electronic tracking device into their care environment would result in greater peace of mind. Peace of mind is a common refrain that coincides with language that sees worry, anxiety, fear, panic, and other psychological and emotional stresses reduced or eliminated. As company 19 stated, “[r]elax and know your loved ones are just a click away. No panic, no worry.” A number of companies also saw their electronic tracking device providing caregivers with more knowledge about their care receiver. This increase in knowledge is related to the various metrics that different electronic tracking devices can track, log, and report. In turn, some companies pointed to data analytics as a way for caregivers, both professional and informal, to “understand how the client is doing” and “establish a baseline of usual, expected behavior” (company 22). These examples helped build toward the aspiration that electronic tracking devices will bolster the relationship between caregiver and person with dementia as well as enhance the ability of caregivers to provide higher-quality care. Finally, some companies saw electronic tracking devices as a way to provide caregivers with opportunities to gain back their own independence through, for example, pursuit of personal interests or moments of respite. As company 12 stated in their company manifesto, “our focus is on ‘freedom’ both for people with dementia and their relatives.”

##### No Ideal World

Although, in many cases, companies had an idealized vision of their products’ impact on stakeholders, some recognized that the future they aspire to is not an ideal world where all worries and vulnerabilities will be duly addressed. Rather, the context and situation will be bounded by the limitations of the devices themselves. The limitations of electronic tracking devices include their role and fit, price, and technological underpinnings. In addition to these, some companies highlighted ethical limitations that persist through device adoption (Table 5).

Electronic tracking devices are envisioned as a powerful tool, aide, and support to caregivers of persons with dementia, capable of providing numerous benefits; however, their role is precisely this—a tool. These devices are not meant to serve as a replacement for the watchful eyes of a human caregiver. Specifying further, many companies were candid about the potential misfit between their device and the needs of a specific caregiver and person with dementia. Not everyone is a good candidate for an electronic tracking device and, although the companies had strived to develop flexible products that could meet any everyday scenario, ultimately, some conceded that other devices may serve caregivers and persons with dementia better; as company 2 stated, “we’re not the only game in town, so don’t hesitate to consider other options.”

The companies were very aware that their target customers (often being on a fixed income) are sensitive to the price of electronic tracking devices. Most companies described their products as being “cost effective” or “affordable,” underscoring the value for price that their package of care tools offered. Coinciding with value propositions, many companies offered resources aimed at helping caregivers procure funding for devices, whether this be through petitioning health insurance companies to reimburse or outright purchase a device, grants from a local charity, or a payment plan.

The worlds that the companies aspire to were often optimistic about the impact of electronic tracking device technologies. This was despite their many limitations. The companies were quick to highlight the technological problems of competitors, with only a few identifying potential limitations within their own choice of technology.

Most companies did not explicitly broach the topic of ethics. A few did explicitly discuss ethics by acknowledging that, despite the benefits that electronic tracking devices bring, ethical conflicts will persist, such as issues related to privacy. On a web page dedicated to “Ethical and technical challenges of GPS transmitters,” company 12 stated the following under the heading “privacy and autonomy”:

The benefit of using GPS transmitters are so great that they to some extent outweigh the disadvantages. The location system may help ensure that the person with dementia does not wander. The main drawback is that most GPS transmitters monitor and record every step the person takes. The person with dementia is therefore monitored 24/7 and not only when needed. So it might be difficult to persuade the person with dementia to use a location system. In addition to concerns over monitoring, people with dementia may often reject things they are not familiar with. Even though you may have gone to a lot of trouble to introduce the location system, some people with dementia will still refuse to have anything to do with it. If this is the case, the best way of showing care is to think about safety rather than having a bad conscience about concealing the location device.

Many companies advised concealing a device on a resistant person with dementia; for instance, company 13 highlighted that its device could easily be slipped into a pocket, stashed in a car, or tucked into a purse or backpack (“don’t embarrass Mom with garish panic jewelry”). Others touted the concealability of their product. Company 12 wrote the following: “In the early stages of Alzheimer’s and dementia, the ability to hide the technology inside a shoe preserves the privacy and dignity of the wearer.”

Despite the emphasis on safety, privacy is still seen as an important value to uphold and, as seen previously, many companies intentionally designed their device to maximize the privacy of persons with dementia while maintaining their safety. Therefore, even though electronic tracking devices have great potential in helping meet the needs of persons with dementia and caregivers, conflicts will remain a problem in the envisioned world of electronic tracking device companies. Another concern is the willful misuse of electronic tracking devices. As mentioned previously, some companies anticipated that their product might be used inappropriately and had taken steps to prevent this. By doing so, these companies defined the boundaries of what they saw as ethically permissible regarding the use of their devices.

#### Characteristics Regarding the Narrative Style of Websites

Next to form and content characteristics, we also found that the manner in which the companies delivered their content could be placed on 3 narrative continuums. The first is between an idealistic and realistic narrative, the second is between a technical and human narrative, and the third is between a company-centric and cocreation narrative.

##### Idealistic to Realistic

Companies using an idealistic narrative spoke about their electronic tracking devices’ capabilities and impact on persons with dementia and caregivers in an idealized manner, using language that did not recognize the real limitations or realities of their electronic tracking devices. For example, company 6 stated that their device had the highest level of accuracy, “ensuring your loved ones and belongings are safe & secure.” When limitations were mentioned, idealistic websites might use hedging language to mitigate perceived impact. Company 13 hedged their device’s reliance on cell networks for high-accuracy location services by saying the following: “When cell service fails [company 13’s device] uses satellite coverage as a back-up, though accuracy may be reduced.”

Limitations in this back-up coverage, nevertheless, remained unmentioned.

On the other side of the continuum, those companies using a realistic narrative provided a more balanced assessment of their products’ strengths, weaknesses, and potential impact on persons with dementia and caregivers. This assessment was distinct from disclaimers or limitations of liability found within contractual documents (eg, terms of service). Company 17 provided a paradigmatic example:

We would like to say there is a 100% guarantee [of locating a missing loved one]; however, there is no such thing, regardless of the technology or method utilized. It must always be remembered you are dealing with people and each situation is different...The best protection is to have trained personnel with the latest equipment available, respond and conduct these searches.

Realistic websites might still portray their devices as being capable of providing great benefit to persons with dementia and caregivers but temper idealized expectations.

##### Technical to Human

The second continuum, technical to human, centers on the approach to addressing the problems of wandering. Websites that used a technical narrative approached wandering as a problem for which technical solutions exist, emphasizing the technical specifications and abilities of their devices as the solution to the problems of wandering. Company 3 is an example of this, with the technical aspects of their electronic tracking device being the center of their marketing content:

[Company 3’s device] provides a way to monitor someone in the home who is at risk of wandering. The system alerts the caregiver when the wanderer goes beyond a set range and provides tracking capability up to 1 mile so that the wanderer can be located and returned.

A human narrative embeds the problems of wandering within the social and relational contexts of persons with dementia and their caregivers. It is not just a technical issue but a human problem. Accordingly, companies that used the human narrative discussed the psychological, relational, and emotional experiences that persons with dementia and caregivers may go through while dealing with wandering and, in this way, acknowledged the complexities of this situation. A clear example of this is the following excerpt from company 1:

Patients with Alzheimer’s and dementia are often frustrated by the worry and concern their families show. Especially in the early stages of memory loss and confusion, families often find themselves struggling with wanting to keep their loved ones safe, while at the same time not stifling their freedom and independence. It’s a difficult and emotional time for everyone involved, but with the Company 1 GPS Safety Device for Dementia you can find the peace of mind you need to help maintain independence for as long as possible without compromising safety.

##### Company-Centric to Cocreation

The final continuum, company-centric to cocreation, centers on how companies articulated some of the elements that contributed to the design process. Company-centric narratives centered on companies bringing their specialized knowledge to bear on the problems of wandering. Whether other stakeholders were included in the process of design was not mentioned. Company 4 stated that “[w]e invented at-risk people tracking in 1986. We started it all. Advantage: Experience.”

On the other end of the continuum lie narratives of cocreation. These companies emphasized that the design process included stakeholders such as persons with dementia, caregivers, older persons, family members, and patient organizations. For example, company 12 repeatedly stated that their product was “[d]eveloped in collaboration with relatives, caregivers and people with dementia.”

## Discussion

### Principal Findings

#### Overview

This is the first study to examine the ethically relevant web-based material of those companies responsible for the design and development of electronic tracking devices. As such, it offers an initial exploration of how those organizations publicly approach ethics in relation to electronic tracking devices in dementia care, revealing that, in general, the companies recognized vulnerabilities facing not only persons with dementia but caregivers as well and, in many cases, acknowledged the 2 as being closely intertwined (eg, bodily vulnerability of persons with dementia contributes to psychological vulnerability). Although the companies’ responses to these needs differed both in care tools offered and values advanced, they affirmed the importance of the safety *and* autonomy of persons with dementia and caregivers’ psychological well-being as values central to their devices. Against this background, 2 specific points are particularly impactful. The first deals with the missing place of persons with dementia within the website content, and the second deals with the presence of a relationship between values and electronic tracking device design.

#### The Place in Content of Persons With Dementia

A major insight from this study is the place persons with dementia occupy within electronic tracking device companies’ website content. Beyond perhaps a few web pages, persons with dementia were largely relegated to the status of third party, being the topic of the conversation rather than a participant. This was evident in the target audience of websites that catered to formal or informal caregivers. For example, the marketing of care tools was from the perspective of what they offered caregivers, that is, how they assisted caregivers’ care practices. This focus on caregivers over persons with dementia is encapsulated in common sentence formulations where the caregiver served as subject and persons with dementia served as related possession—a care tool benefits *you* (ie, caregiver) and *your* person with dementia, as seen, for example, in the following quotation from company 9 (emphasis ours): “Designed to help *you* and *your* loved one feel safe, in control and have peace of mind.”

This conclusion builds upon previous research—which found no person with dementia–centric marketing content within retail websites based in the Netherlands, the United Kingdom, and Sweden [17]—by demonstrating that the relegation of persons with dementia to a third party occurs at the level of organizations responsible for the development of electronic tracking devices. That companies do not create content directed at persons with dementia is striking given the larger push in dementia care to create opportunities of empowerment for persons with dementia through care practices, shared and supported decision-making models, and a movement toward person-centered care [60]. Even though the companies touted their products as enhancing the autonomy and freedom of persons with dementia while maintaining their safety, the actual choice to use these devices appears to be completely left out of their hands and placed solely within the purview of caregivers. This also raises questions regarding the role of companies in the informed consent process, particularly when selling directly to informal caregivers.

In addition to being a third party within the marketing material, a close examination of the offered care tools shows that most tools acted upon persons with dementia while largely empowering caregivers. With the notable exception of those devices that offered SOS functionality or person with dementia–initiated phone calls, most tools involved caregivers acting upon persons with dementia, for instance, one-way listening or tracking metrics such as location. This emphasis on caregivers may lead to questions about who this device is truly meant for, contributing to the lack of consensus on the nature of electronic tracking devices, which have been suggested to be a form of assistive technology [61], surveillance technology [62], telecare [63], smart wearables [42], and monitoring technology [64], among others.

It is known that persons with dementia have a strong desire to maintain their autonomy in decision-making concerning electronic tracking devices [65,66]. In this regard, a key takeaway from this study is that electronic tracking device companies should invest in making their marketing more inclusive of persons with dementia by centering their focus on the relationship between persons with dementia and caregivers. This reorientation toward the relational both brings persons with dementia into the conversation about their own future care and bolsters the relationships that undergird and enable this very autonomy [58]. Thus, rather than simply moving content focus from one artificially isolated audience to another, a more accurate picture of the real-life relational context in which decisions about electronic tracking devices occur is presented. This relational context is evident, albeit in truncated form, within the identification of interrelated vulnerabilities (eg, bodily and psychological) and within those websites using a human narrative as they often emphasized the very real relational and emotional conflicts that caregivers experience in wanting to balance the safety and autonomy of the person with dementia in their care. Furthermore, a move toward the relational dimension would greatly benefit the pursuit of person-centered care as it incorporates relational ethics within itself [67].

#### Values and Electronic Tracking Device Design

An additional insight pertains to the arc from identified vulnerabilities to care tools to a world aspired to. It is evident that the companies linked particular values to design decisions. For example, those companies putting forward electronic tracking devices that provided a medium level of information availability stressed the privacy of persons with dementia as a core value that should be placed above others such as constant information availability. Such decisions to place relative importance on certain values was found in the other levels as well. Companies with low level of information availability stressed reliability and dependability, and those with a high level of information availability stressed information, situational awareness, and control of care. In deliberately choosing to uphold certain normative values, the companies also made the decision (whether explicitly or implicitly) to not address or to de-emphasize other values. Thus, to uphold privacy requires a sacrifice in the amount of real-time information available to caregivers, and to uphold maximum information availability requires a sacrifice in the privacy of persons with dementia. Companies are choosing to give weight to certain values and link them to product design elements and, therefore, their devices are intrinsically value-laden artifacts.

The connection between design decision-making and particular values is not surprising given the abundance of literature focusing on the ethics of design. Indeed, a diversity of theories and methods has been put forward to infuse ethics into the design process—among others, *value-sensitive* design [28,68,69]*, responsible research and innovation* [70,71]*, reflective design* [30], *constructive technology assessment* [72]*,* and *technology mediation* [73].

#### How Do Values Emerge Within Electronic Tracking Devices

Knowing that companies link values to design, an important question to ask is how, exactly, do these particular values come to be embraced? Some preliminary insights can be gleaned from electronic tracking device company websites. First, it is evident from those companies using a narrative of “co-creation” that stakeholders are involved in the process of development in some capacity. Second, engagement with academic research is also present. Most notably, company 2 had a web page with links to relevant academic publications, and company 12 repeatedly highlighted its collaboration with academic institutions (being a public-private endeavor). An additional indicator of academic engagement is the large overlap in company-identified vulnerabilities and values that are also present within academic literature. Company emphasis on caregiver conflict between balancing the aim to keep a person with dementia safe with the wish to maintain a person with dementia’s autonomy, for instance, reflects the central conflict most preoccupying the normative literature regarding electronic tracking devices in dementia care [18].

Although these early insights demonstrate that some companies engaged with stakeholders and the academic literature in the process of bringing their product to market, it is premature to draw too detailed conclusions regarding the link between value and design. The internal processes that gave rise to the company website content remain opaque and, as such, many questions are left unanswered. For example, it cannot be said that those companies that did not mention stakeholder involvement on their website excluded stakeholders entirely from the design process. Similarly, it is unknowable from the websites who made decisions or when, in the course of development, they were made (eg, were they a decision of design or post hoc marketing strategy?).

### Future Research Orientations

However, what these insights do offer is a way to orient future research. It is clear that the companies identified a series of vulnerabilities facing persons with dementia and caregivers as a result of wandering and that, in crafting devices to address these vulnerabilities, they identified certain care tools and values deemed necessary to bring about a certain conception of life. Future research should seek to better understand both the internal company processes that give rise to certain value decisions and the perceptions of electronic tracking device developers regarding the ethics of electronic tracking device design, development, and use. The former may uncover the extent to which stakeholders are involved in the development process, including who developers consider to be relevant stakeholders as well as the formal use of any approaches to design ethics, and the latter may increase understanding of how developers conceive autonomy, personhood, and the lived reality of persons with dementia—all important factors that contribute toward the construction of the envisaged user of their device (eg, whether idealized or realistic [74]) and the values developers design for. Undertaking such research would reveal much about how companies navigate the ethical landscape that their products will operate within [73], ultimately contributing to greater harmony between industry, health care professionals, persons with dementia, and informal caregivers.

### Strengths and Limitations

This study is the first to provide a nuanced analysis of electronic tracking device companies’ website content, showing how organizations responsible for the design and development of electronic tracking devices portray their products within the context of dementia-related wandering. The strength of these results is bolstered by the study’s strong methodological rigor. First, the process to identify websites used a particularly robust methodology, which is a multipronged strategy based on the PRISMA guidelines. Second, we used a method that preserved the websites in their entirety as they were at a particular time, mitigating the known data preservation problem associated with internet-based research [52]. Not only did this allow for a uniform and constant data collection process, but it also provided an opportunity to dive deeper into websites to ensure all relevant data were extracted without fear of data either disappearing or being altered. The richness of the data collected from this process benefited from reproducible procedures of the QUAGOL data analysis methods, which necessitate prolonged engagement with the data. From its initial full reading to the creation of individual schemes and then merged schemes, the QUAGOL requires a researcher to consistently engage with and to return to the original data. This data analysis process is traceable via an audit trail of conceptual schemes, researcher journals, and meeting notes (Multimedia Appendix 2). Third, data analysis and synthesis involved 3 researchers performing independent analysis at certain points with a high degree of consensus, strengthening the reflexivity and rigor of the results reported.

There are a few limitations to this study. First, only accepting English-language websites introduces a potential risk of bias and reduces potential transferability of the results. Second, because of the nature of the websites, the web pages included in this study may have since undergone significant changes or become defunct. Any in-depth research of websites will potentially be a step behind as the data are artificially frozen in time. Third, as indicated in the discussion, there is a limit to the complexity and depth of the results that can be derived from company websites.

### Conclusions

Although persons with dementia are the focus of electronic tracking device use, they are not the focus of electronic tracking device company websites. Website material is more akin to a conversation between companies and caregivers about persons with dementia than with persons with dementia. This relegation of persons with dementia to a third party is an important conclusion, one that should serve not only as a sign that electronic tracking device companies should focus on developing materials centered on the relationship between caregivers and persons with dementia but perhaps also as a starting point for critical reflection on the enterprise that gives rise to these devices and the level of person with dementia involvement in their design, development, and use. In particular, serious reflection should be given to the question of how persons with dementia can be further involved in the use of electronic tracking devices when they are presently not a focus of marketing material. Indeed, a second important conclusion is the presence of three conceptual categories that form a triptych (ie, they are closely associated and better appreciated as a whole): identified vulnerabilities, care tools, and a world aspired to. As presented, vulnerabilities facing persons with dementia and caregivers are addressed through specific care tools by means of bringing a particular world to life after electronic tracking device implementation. These 3 concepts are further linked, in some fashion, to stakeholders; academic research; and, most vitally, values. What remains unknown is how these conceptions come to be. How are vulnerabilities identified? What process has led to the decision to center an electronic tracking device on a particular value such as privacy? By what means are stakeholders or the results of academic research included in this process? Future research should turn to understanding how the content of electronic tracking device company websites came to fruition. Doing so would provide a deeper foundation for the ethical evaluation of electronic tracking devices used in dementia care.

## Appendix

Multimedia Appendix 1 Data collection form used to collect company, electronic tracking device, and website characteristics.

Multimedia Appendix 2 Examples of an early and later conceptual scheme developed during data analysis.

Multimedia Appendix 3 Table of electronic tracking device characteristics from the included websites.

## Abbreviations

PRISMA: Preferred Reporting Items for Systematic Reviews and Meta-Analyses
QUAGOL: Qualitative Analysis Guide of Leuven
