# Supplementary material for: Electronic Tracking Devices for People With Dementia: Content Analysis of Company Websites
Source: JMIR Aging. 2022 Nov 11;5(4):e38865. doi: 10.2196/38865 (PMC9700241; doi:10.2196/38865)
Supplement: Multimedia Appendix 1 [file aging_v5i4e38865_app1.pdf]

File: Supplemental material 1

Title: Electronic tracking devices for people with dementia: A content analysis of company websites.

Journal: JMIR Aging

Description: This is a Multimedia Appendix to a full manuscript published in the J Med Internet Res. Below is a data collection form used to collect company, electronic tracking device, and website characteristics.

Content Analysis Characteristics

- Name of Company:
- Country of Company:
- Company Structure (i.e., for profit or non-profit):
- Target audience:

- ETD
  - Language used to describe ETD:
  - Definition of ETD used:
  - Technology used in ETD:
  - Number of ETDs on sale:
  - Price of devices:

- Type of website content:

Multimedia (Audiovisual)	Textual

- What are the target users of these devices?
