# Supplementary material for: Electronic Tracking Devices for People With Dementia: Content Analysis of Company Websites
Source: JMIR Aging. 2022 Nov 11;5(4):e38865. doi: 10.2196/38865 (PMC9700241; doi:10.2196/38865)
Supplement: Multimedia Appendix 2 [file aging_v5i4e38865_app2.pdf]

File: Supplemental material 2

Title: Electronic tracking devices for people with dementia: A content analysis of company websites.

Journal: JMIR Aging

Description: This is a Multimedia Appendix to a full manuscript published in the J Med Internet Res**.** Below is an example of an early individual conceptual scheme developed during data analysis.

Company 1 Short Report: April 06, 2021

Initial thoughts: The content and word choice that Company 1 uses seems to revolve around certain key themes or ideas: Safety, protection, independence (PWD), peace of mind, knowledge.

Again and again the notion of maximum protection is mentioned. Caregivers want to provide the best care possible, they want to ensure PWD are protected from danger and kept safe. A part of this is *knowing* that a PWD is safe (through use of ETD tools). The expected results are that the caregiver gains peace of mind, knowing that the PWD is safe. The PWD gains/maintains/extends independence (or at least a feeling of independence), which depending on your conception of autonomy might still be, true, independence, and they have an increase in safety if a wandering event occurs.

For public safety agencies, the emphasis is placed on proactivity: improving response time, broadening safety readiness, preventing tragedy, and lowering departmental cost via reducing repeat calls/episodes of wandering.

NOTES:

Needs/vulnerabilities of PWD:

1. PWD are unable to protect themselves, PWD are vulnerable to being harmed during wandering.
2. Maintaining independence is important for PWD.
3. PWD can get frustrated/anxious if caregiver wants to accompany them all the time/restrict independence (emotional/psychological stress?).

Needs/vulnerabilities of Caregivers:

4. Caregivers anxious/worried about well being of PWD (Emotional/psychological stress).
    1. Want to *know* that PWD are safe! Knowledge is important!!
5. Want to protect and keep PWD as possible (Duty to care?)

How Company 1 ETD responds:

6. Allows caregivers to use a variety of tools to track, locate, and review the location history of PWD.
    1. *Maximizes* safety - quick recovery, knowing when PWD are not on schedule, etc.
        a. Maintain PWD's independence longer

   b. Ease anxiety/stress of caregiver
7. Company 1 provides more protection than other devices not designed for PWD/vulnerable people.
   1. By providing a suite of safety features
      a. That proactively detects unsafe situations, thereby preventing emergencies.

Key focus: Balancing the need to keep PWD safe vs. Not stifling their independence. (i.e., Beneficene/non-maleficence vs. Autonomy --> as seen in ethics literature as well!). Company 1 maximizes safety and protection while also maximizing/extending PWD's independence.

Description: Below is a late stage example of the same individual conceptual scheme further refined during data analysis.

Company 1 Conceptual Scheme: May 21, 2021.

Vulnerabilities

- PWD
  - Physical Vulnerability
    - PWD at every stage of dementia are vulnerable to wandering, getting lost, or becoming disoriented. (Wandering = risk of serious injury/death).
    - Majority of PWD (6 in 10) are vulnerable to wandering à putting their life at risk.
    - PWD (elderly) are vulnerable to falls, subsequently not being able to get up, or reach phone for help (physical harm resulting from inability to seek help).
    - Longer time elapses from when a PWD is lost = higher risk of serious injury or death.
    - PWD are vulnerable both in and out of home.
  - Existential
    - PWD are vulnerable to losing their independence, losing their quality of life. (Discusses independence as related to QOL, and how PWD do not want to give up their QOL/independence, I think a little more substantial).
  - Psychological/Emotional vulnerability
    - PWD have limited safety awareness.
    - PWD are vulnerable to hard/inconvenient to wear technology (frustration/anger/anxiety when wearing other non-company 1 ETDS).
    - PWD are vulnerable to feeling unsecure/safe ("should enjoy feeling of security")
    - PWD are vulnerable to losing their independence
  - Relational vulnerability

        - Vulnerable to losing connection/communication with caregivers.
- Caregivers
    - Psychological/Emotional Vulnerability
        - Psychological/emotional stress due to worrying about PWD safety/well being.
        - Vulnerable to not knowing if PWD are safe/where they are à fear; terrifying situation.
        - Vulnerable to unreliable or difficult to use technology. ?? (Maybe somet other category?)
    - Relational Vulnerability
        - Connection/communication with PWD is vulnerable.
    - Moral
        - Want to keep loved ones safe but not stifle PWD's freedom and independence.
        - When PWD are still capable of doing daily tasks it can be difficult to put limitations on them (out of fear for their safety).

<u>Features of ETD that address vulnerabilities (how does ETD provide care tools?)</u>

- Design
    - Ease of Use
    - Comfortable to wear
    - Discreet.
    - Easy to use application (ios, android, website) for reviewing location/other information, setting up safe (geo-fence) zones, operating ETD, etc.
- Information
    - Bolsters Knowledge
    - Notifications sent when PWD leaves safe areas, enters unknown areas, is late/off schedule, arrives at destinations.
    - Keeps detailed history of transits and locations.
    - Continuous, all-day tracking
    - Safeguard GPS — Accurate and reliable.

- Communication
    - 2-way conversation (like a mobile phone)
    - 1-way "listening" (allow caregiver to listen in to surroundings of PWD)
    - SOS Button

<u>World Aspired To</u>

PWD

- Bodily dimension
    - Maximum safety for PWD/Provide maximum protection
- Psychological
    - PWD independence for longer (as long as possible without compromising safety).
        - Extended quality of life –maintain independence
    - PWD can feel independent

Caregiver

- Psychological

  - Give caregivers peace of mind
  - Caregivers *know* where PWD are at all times.
    - Allow caregivers to be there when PWD needs them (know something is wrong and respond accordingly).
    - Allow stronger/deeper connection between PWD and caregiver
    - Allow PWD independence while not compromising their safety.
  - Not burden by technological solutions
- Relational
  - Deeper connection with PWD.

Thoughts:

The world company 1 is striving for is one where PWD receive maximum protection/safety against the risk/dangers of wandering, while also enjoying independence longer. Caregivers receive greater peace of mind, from knowing 1) the location of their loved one at all times and 2) that they can be found quickly if wandering occurs. Related to this, is the notion that the ETD is easy to use, easy to integrate into life, and therefore doesn't pose an added burden to caregivers. Ultimately, this all adds up to deeper connection/communication between PWD and their caregiver.

<u>Continuum:</u>

1) Idealistic — Uses more idealistic language, although certain hedging language is present. E.g. "ensure safety", "complete safety solution for dementia" "always know they (PWD) are safe" vs. "provide maximum protection" (Maximum protection is more hedging-like).

2) Human narrative — Rather than focusing on the problem of wandering as an isolated problem requiring technical solution, company 1 places wandering within the context of the relations between caregiver and PWD. The ETD enables the relationships, habits, and lifestyle of PWD to continue while also easing the psychological/emotional stress that the caregiver experiences as a result of anxiety/worry/etc. about PWD possibly wandering.

3) Company-Centric — I couldn't find anything that indicates that the device is made with stakeholders of dementia wandering. This makes some sense, given the ETD was originally designed for special needs children.

4) Product — Has a "Customer care team" that comes with the subscription, but this seems to be more standard customer service rather than a robust response/emergency call center. Therefore, I categorize this as just a product, as it is meant to be used by a caregiver without any interaction with the company (in the sense that if nothing goes wrong with tech, the caregiver would never need to contact company beyond paying monthly bill).

<u>Golden Circle</u>

What: Need to maintain independence and safety (PWD), and reduce psych/emotional stress (caregiver)

How: ETD that easily integrates into PWD/Caregiver life, that provides enough information to a caregiver so they know a PWD is safe, and to empower them to intervene in a wandering event.

Why: To bolster a caregiver's ability to grant PWD greater independence by maximizing PWD safety by strengthening connection between PWD and caregiver (through information?).
