# Supplementary material for: Electronic Tracking Devices for People With Dementia: Content Analysis of Company Websites
Source: JMIR Aging. 2022 Nov 11;5(4):e38865. doi: 10.2196/38865 (PMC9700241; doi:10.2196/38865)
Supplement: Multimedia Appendix 3 [file aging_v5i4e38865_app3.pdf]

File: Supplemental Table 1

Title: Electronic tracking devices for people with dementia: A content analysis of company websites.

Journal: JMIR Aging

Description: This is a Multimedia Appendix to a full manuscript published in the J Med Internet Res. Below is a table of the characteristics of ETDs found within included websites.

Supplementary Table 1: ETD characteristics

Characteristic	Number of companies (N=29)*
Device form factor	
Fob/Tag	14
Watch	5
Smartphone application	2
Shoe sole	1
Wrist band	6
Technology Used	
GPS + Mobile network (+WIFI, Bluetooth)	22
Radio frequency	7
Price	
Device price	
Free tier	2
$1-$200	13
$200-$800+	10
Subscription Price†	
$1-$50 per month	19
$50-$100 per month	3
No subscription required	4
Pricing not available on website	6

*Companies may have multiple characteristics.

†Subscriptions may include: Mobile SIM plan (voice/data), 24/7 response call center, customer service/support, expanded ETD features, access to mobile application or online web portal. ETDs that do not require a subscription can be operated without additional monthly fees, though may require replacement of batteries, wrist bands or other parts. All denominations were converted to the US dollar.
